# Simultaneous Occurrence of Medullary Thyroid Carcinoma and Papillary Thyroid Carcinoma: A Case Series with Literature Review

**DOI:** 10.3390/curroncol30120745

**Published:** 2023-11-30

**Authors:** Poupak Fallahi, Armando Patrizio, Giulio Stoppini, Giusy Elia, Francesca Ragusa, Sabrina Rosaria Paparo, Eugenia Balestri, Valeria Mazzi, Chiara Botrini, Gilda Varricchi, Salvatore Ulisse, Marco Ghionzoli, Alessandro Antonelli, Silvia Martina Ferrari

**Affiliations:** 1 Department of Translational Research and New Technologies in Medicine and Surgery, University of Pisa, 56126 Pisa, Italy; poupak.fallahi@unipi.it (P.F.); s.paparo@studenti.unipi.it (S.R.P.); 2 Department of Emergency Medicine, Azienda Ospedaliero-Universitaria Pisana, 56126 Pisa, Italy; armando.patrizio@ao-pisa.toscana.it; 3 Department of Surgery, Medical and Molecular Pathology and Critical Area, University of Pisa, 56126 Pisa, Italy; giulio.stoppini@yahoo.it (G.S.); giusy.elia@phd.unipi.it (G.E.); francesca.ragusa@phd.unipi.it (F.R.); e.balestri7@studenti.unipi.it (E.B.); valeria.mazzi@ao-pisa.toscana.it (V.M.); chiara.botrini@med.unipi.it (C.B.); 4 Department of Translational Medical Sciences, Center for Basic and Clinical Immunology Research (CISI), World Allergy Organization (WAO), Center of Excellence, Institute of Experimental Endocrinology and Oncology (IEOS), National Research Council, University of Naples “Federico II”, 80138 Naples, Italy; gilda.varricchi@unina.it; 5 Department of Surgical Sciences, ‘Sapienza’ University of Rome, 00161 Rome, Italy; salvatore.ulisse@uniroma1.it; 6 Division of Pediatric Surgery, Department of Surgical Pathology, University of Pisa, 56126 Pisa, Italy; marco.ghionzoli@unipi.it; 7 Department of Clinical and Experimental Medicine, University of Pisa, 56126 Pisa, Italy; silvia.ferrari@unipi.it

**Keywords:** papillary thyroid cancer, medullary thyroid cancer, simultaneous cancers, RET mutation, thyroid ultrasound

## Abstract

Background: Papillary thyroid carcinoma (PTC) is the most common type of differentiated TC, while medullary TC (MTC) accounts for 4%. The concomitant presence of PTC and MTC is rare. Methods: This is a retrospective, single-center observational study conducted over 16 years (2001–2017). The data were collected from the clinical records of patients who underwent total thyroidectomy at the Endocrine Unit-Department of Medicine of the University Hospital of Pisa, Italy. Results: Over 690 analyzed cases, 650 (94.2%) were exclusive DTC, 19 exclusive MTC (2.75%) and 5 PTC/MTC (0.7%). No case of mixed medullary/follicular TC or hereditary MTC (familial MTC/multiple endocrine neoplasia type 2) was found. Among the five PTC/MTC cases, there was a male prevalence (M:F = 3:2), and all PTC components were at stage I, whereas 40% of MTC were at stage I and III and 20% of MTC were at stage II; microPTC (mPTC) was prevalent (80%) and also microMTCs were frequent (40%); 60% of MTC patients recovered, while 40% of patients developed metastatic disease. The search for germline mutations of the RET gene resulted in being negative in all cases. Conclusions: The incidence of PTC/MTC has been increasing over the past 30 years. The etiology of PTC/MTC forms is still unknown, and although this simultaneous occurrence could be only a coincidence, we cannot exclude the hypothesis of a shared genetic origin.

## 1. Introduction

Thyroid malignancies are the most frequent neoplasms of the endocrine system. Approximately 95% of thyroid malignancies are differentiated thyroid carcinomas (DTCs) arising from follicular cells (papillary or follicular carcinomas) [1,2,3]. Papillary thyroid carcinoma (PTC) is the most common type of DTC, accounting for approximately 85% of them. It is a slow-growing tumour that may remain localized for years. It characteristically metastasizes first to cervical lymph nodes. Microscopic foci of papillary carcinoma are common at autopsy and thought to be present in as many as 30% of adults [1]. Medullary carcinoma of the thyroid (MTC) accounts for 4% of all thyroid cancers (TCs), and it arises from neuroendocrine C cells (parafollicular cells) [1,2]. Approximately 75% of medullary carcinomas are sporadic and 25% hereditary. MTC is also a component of multiple endocrine neoplasia (MEN) types 2A and 2B. Of note, familial MTC syndrome (FMTC), characterized by MTC without hyperparathyroidism and pheochromocytoma, is now considered a variant of MEN 2. These syndromes are caused by mutations of different regions of the RET proto-oncogene [1,2]. The concomitant presence of PTC and MTC is rare [3]. The presentation pattern can be of two types: mixed medullary/follicular thyroid carcinoma (MMFTC), in which the two components are present within the same neoplastic nodule, or PTC/MTC, in which the two components are separated from healthy thyroid tissue [4]. Many in the scientific community believe that this rare form of TC is just the random development of two independent cancers entities. However, it is not yet possible to completely exclude the hypothesis of a common origin of the two neoplasms, and the phenomenon still deserves to be investigated [3,5]. The aim of the current case series is to describe the epidemiological characteristics, disease conditions and clinical outcome of five patients with simultaneous PTC/MTC encountered at our center and to compare the results with the updated data reported in the scientific literature.

## 2. Materials and Methods

This is a retrospective, single-center observational study conducted over 16 years (from 2001 to 2017). The data were collected from the medical records of patients who consecutively underwent total thyroidectomy at the Endocrine Unit-Department of Medicine of the University Hospital of Pisa (AOUP), Italy. The participants were enrolled in consecutive order. No other inclusion/exclusion criteria were considered. For both PTC and MTC, the TNM 8th edition staging system was applied [6]. Germline RET gene mutation data were collected (Sanger sequencing) on blood samples, with most common involved exons (5, 8, 10, 11, 13, 14, 15, 16) being tested. We then performed a literature search of MEDLINE/PubMed databases regarding simultaneous PTC and MTC published since 2000 up to 31 January 2023. We included original articles, reviews, viewpoints, commentaries, case series and case reports. The search terms, used both separately and in combination, included: “DTC”, “PTC”, “MTC”, “thyroid cancer”, “medullary thyroid cancer”, “differentiated thyroid cancer”, “papillary thyroid cancer”, “synchronous”, “simultaneous” and “cooccurrence”. Articles published in languages other than English were excluded. This paper has been written in line with the PROCESS 2020 guidelines [7]. Descriptive statistics were performed.

## 3. Case Presentations 

### 3.1. Case 1

A 59-year-old man without a family history of thyroid disease and suffering from chronic lymphocytic thyroiditis (CLT) was submitted to fine needle aspiration cytology (FNAC) of a middle-pole left lobe thyroid nodule (EU-TIRADS 5 [8]). It measured at the neck ultrasound (US) 4.3 × 2.4 × 1.6 cm, which resulted in malignant cells suggestive of MTC. Laboratory tests showed elevated calcitonin (Ct) (>894 pg/mL, normal range 0–11.5 pg/mL) and carcinoembryonic antigen (CEA) (22.2 ng/mL, normal range 0–5 ng/mL). Thus, he was submitted to total thyroidectomy and lymphadenectomy of the central cervical compartment. The histological report demonstrated the presence in the left lobe of 1.3 cm MTC and, in the right lobe, of CLT with a papillary thyroid microcarcinoma (mPTC), follicular variant, of 0.2 cm. The patient also had a single cervical lymph node (level VI-left) positive for MTC metastasis; therefore, he was classified as TNM stage I for PTC and stage III for MTC [4]. Germline RET mutations on peripheral blood cells turned negative, whereas somatic mutations analysis was not performed. The patient is currently being followed up for 15 years with biochemical evidence of cured diseases (last Ct and thyroglobulin (Tg) values <2 pg/mL and <1 ng/mL, respectively). 

### 3.2. Case 2

A 55-year-old woman with a positive family history of thyroid disease and affected by multinodular goiter (MNG) showed a hypoechoic nodule of 0.6 × 0.6 × 0.6 cm on the middle-lower pole of the left lobe, with perinodular and poorly central vascularization (EU-TIRADS 4) and reactive bilateral lymph nodes on neck US. Circulating Ct was quite above the normal range (16.9 pg/mL). Thus, FNAC was performed along with the calcitonin level on the aspirated sample fluid, which turned extremely high (>2000 pg/mL). Subsequent total thyroidectomy and emptying of the compartment central cervical lymph node demonstrated the presence of a micro-MTC (mMTC) of 0.6 cm infiltrating the parenchyma and of a classic variant mPTC of 0.4 cm in the left thyroid lobe, with no lymph nodes metastasis (TNM stage I for both PTC and MTC). Routine testing for germline RET mutations was negative. At the last 4-year follow up, the patient had both normal calcitonin and thyroglobulin (Ct < 2 pg /mL and Tg < 1 ng/mL).

### 3.3. Case 3

The thyroid US of a 56-year-old man with MNG showed on the upper pole of the left lobe a slightly hypoechoic nodule of 0.9 × 0.9 × 0.8 cm (EU-TIRADS 4), whose cytological examination after FNAC was suspicious for malignant cells, and the BRAF V600E mutation was detected in the cytological sample. The blood Ct value was normal (6.3 pg/mL). After total thyroidectomy, the pathology report demonstrated the presence of a 0.2 cm mMTC in the right lobe and classic variant PTC of 1.1 cm in the left lobe. The patient’s TNM stage was I for both diseases. Routine germline RET mutations in blood cells resulted in being negative. At the last visit, 5 years from the diagnosis, the patient had both Tg and Ct undetectable.

### 3.4. Case 4

After fourteen years from a right subtotal thyroidectomy, performed in a different institution and resulting in an MTC of 2.8 cm (stage II), a 66-year-old woman showed a new significant increase in blood Ct measurement to 478.0 pg/mL. She was then subjected to a positron emission tomography/computed tomography (PET/CT) scan with 2-deoxy-2-[fluorine-18] fluoro-D-glucose (18F-FDG), which, however, did not demonstrate sites of metabolic activity. The neck US instead showed a solid hypoechoic nodule in the right lobe with microcalcifications (which measured 1.7 × 0.9 × 0.8 cm) that led the patient to a completion thyroidectomy together with the removal of the central compartment of the lymph nodes. A left lobe 0.1 cm mPTC with two lymph nodes positive for MTC metastasis (Level VI) was detected (TNM stage I for PTC and stage III for MTC). A routine test for germline mutations of RET on blood cells was found negative. A subsequent PET/CT scan with a different tracer, the 3,4-dihydroxy-6-(18)F-fluoro-l-phenylalanine (18)F-FDOPA), disclosed metabolic activity of a very small lymph node at the right lateral cervical chain (II level), not evident at neck US, whereas the Ct and CEA values were above normal values (274 pg/mL and 6.3 ng/mL, respectively), indicative of relapsing and metastatic MTC. Based on the minimal burden of disease, a conservative approach was adopted and the patient was followed up every 6 months with stability at the neck US and with slowly waning the Ct levels until the value of 1.8 pg/mL after 21 months from the second surgery, reaching the status of biochemically cured. Then, the patient was lost to the follow-up.

### 3.5. Case 5

A 41-year-old man suffering from MNG underwent neck US, showing bilateral hypoechoic nodules with intranodular vascular spots. The basal Ct was very high (2.474 pg/mL). The following FNAC of a 45 mm left nodule (EU-TIRADS 5) turned malignant and a total thyroidectomy with central neck dissection was performed. The histology report resulted in right lobe 0.4 cm mPTC and left lobe 4.7 cm MTC infiltrating the capsule with surrounding normal parenchyma. The lymph nodes examination was negative for metastasis (TNM stage I for PTC and stage II for MTC); germline RET mutations in blood cells were negative. After three consecutive years of undetectable levels, the Ct values increased to 44.9 pg/mL and the patient was thus submitted to a PET/CT scan with 18F-FDOPA showing high tissue metabolic activity at the right latero-cervical region, consisting of MTC metastasis and managed with surgery. Nevertheless, the levels of Ct kept increasing steadily to 276 pg/mL, new lymph nodes metastases were identified through US and PET and, after 1 year, a third surgery was performed. Nine months later, considering the progressive and the unresectable locally advanced disease (Ct 1114 pg/mL, evidence of disease at the right lateral neck region), systemic therapy with multitargeted kinase inhibitor (MKI) vandetanib 300 mg/day was started with good tolerance by the patient. After two months, the dose of MKI was reduced to 200 mg/day because of a prolonged QT interval at EKG and, later, for persistence of the condition, was switched to cabozantinib 60 mg/day. At the last follow-up visit, six years from the first thyroid surgery, the patient showed persistent, asymptomatic small-volume locoregional MTC (last Ct, 3295 pg/mL).

## 4. Results

The medical records of 690 cases of TC were examined. Among them, 650 (94.2%) patients had a diagnosis of exclusive DTC, 19 patients with exclusive MTC (2.75%) and 5 cases of PTC/MTC (0.7%). The remaining cases were represented by undifferentiated thyroid carcinomas and non-thyroid cell neoplasia. Of five cases of PTC/MTC, there was no case of MMFTC or hereditary MTC (FMTC or MEN2). The prevalence of PTC out of all cases of MTC (19 exclusive and 5 simultaneous with PTC) was 20.8% (5/24). The prevalence of exclusive MTC out of all cases of DTC (650 exclusive and 5 simultaneous with PTC) was 2.9% (19/655). Our final case series included five cases, comprising two (40%) females and three (60%) males. The average age of diagnosis was 55.4 years. The first diagnosis in four of five patients (80%) was of MTC suspected by elevated pre-operative levels of circulating serum calcitonin. Table 1 lists the details for each case (see Table 1). 

In these five cases, there was no MMFTC nor hereditary MTC (familiar medullary thyroid cancer or associated to multiple endocrine syndromes). We found a prevalence of the male gender (60%), the ratio being M:F = 3:2. All cases had an underlying thyroid disease (three MNG, one multinodular thyroid disease and one CLT) and, in four cases out of five, there was a family history of thyroid disease. Among the biopsied nodules, two had ultrasonographic characteristics consisting of an EU-TIRADS score of 4 and the other two had an EU-TIRADS score of 5. The average size of MTC was 1.92 cm. The average size of PTC was 0.42 cm. The prevalence of mPTC in concurrent cancers was 80% (4/5), and one patient (case 3) had a classic variant PTC component with dimensions of 1.1 cm. The prevalence of mMTC in concurrent cancers was 40% (2/5). The histological variants found in PTCs were 2/5 classic, 1/5 follicular and 2/5 non specified. At the diagnosis, all PTCs were at stage I (100%), whereas the MTC counterparts were at stage I and III in 2/5 each (40%) and at stage II in 1/5 patient (20%). The search for germline mutations of the RET gene was conducted in all five patients and they all tested negative. The search for BRAF mutations, on the PTC finding of FNAB, was conducted in a single patient (case 3) and tested positive. During the follow-up period, which lasted from 4 years (patient 2) to 15 years (patient 1 and 4), 4/5 (80%) MTC patients were considered biochemically cured, while 1/5 (10%) patients developed loco-regional advanced disease, needing several surgeries and finally systemic therapy with MKI; no recurrence of PTC was detected. 

## 5. Review of the Literature

Regarding our literature review, we found 22 papers published between 2000 and the end of January 2023 that satisfied our search criteria, and that consist of 468 cases of simultaneous DTC and MTC [3,5,9,10,11,12,13,14,15,16,17,18,19,20,21,22,23,24,25,26,27,28]. We summarize the cases in Table 2 (see Table 2). When available, the majority of DTC counterparts consisted of mPTC, as confirmed by some of the largest series (i.e., prevalence of mPTC of 81% [3] and 78% [22]), and most reported cases displayed well-separated MTC and PTC components, which may have a different significance to MMFTC. The genetic analysis of the RET status was investigated in 14/22 (63%) of the considered studies, even if it was not performed in all patients present in each series, especially in those including the largest numbers of patients (200/468, 42%): of these, 19/200 (9.5%) harbored germinal mutations. The search for other genetic mutations typically associated with TC was reported in only 6/22 (27%) papers and 29/468 (6%) patients: 5/29 (17%) cases carried BRAF V600E mutation, 1/29 (3%) H-RAS and 1/29 (3%) K-RAS, with negative results in 22/29 (75%).

## 6. Discussion

The simultaneous occurrence of papillary and medullary carcinoma in the same thyroid gland is a rare but noteworthy condition. In the literature, the prevalence of this simultaneous occurrence encompasses 19% of all cases of MTC [13] and 0.28% of all cases of DTC [19]. The pattern of presentation can be of two types: MMFTC, in which the two components are present within the same neoplastic nodule, or PTC/MTC, in which the two components are separated by thyroid tissue. The histological findings of PTC/MTC do not differ from those of the isolated PTC and MTC forms, while the MMFTC form can be present in at least three ways: MTC and follicular cell hyperplasia, MTC and PTC and MTC and DTC with cells positive for both calcitonin and thyroglobulin at immunostaining evaluation [29]. The etiology of the PTC/MTC forms is still unknown although many believe that this simultaneous occurrence is only a coincidence (“collision theory”). Hypotheses of a non-random origin of PTC/MTC forms take into account the existence of a common cellular progenitor, a common carcinogenic stimulus (“field effect theory”) or common molecular mutations [18,30]. To date, it has been excluded that the classic mutations involving RET, BRAF and RAS oncogenes found in cases of exclusive PTC and MTC could be the basis of PTC/MTC [22]. In our series, none of the five patients examined had germline RET mutations and only one patient was investigated and found to have a BRAF mutation in the PTC counterpart. The incidence of PTC/MTC has been increasing over the past 30 years. This rise is only in part explained by the augmented incidence of mPTC and PTC in the population and by the improvement of modern diagnostic techniques [16]. MTC in combination with PTC presents predominantly hypoechoic or very hypoechoic nodules and microcalcifications on US, which is similar to the other DTC [31]. The dosage of Ct was performed in all patients at the time of the FNAC, as per our center’s protocol, and it was elevated in 80% (4/5) of the cases described here. Thus, this approach resulted in being very useful for the identification of MTC, since neither ultrasonography nor cytological analysis are generally conclusive for distinguishing the different subtypes of cancer [32]. In fact, only in cases 1 and 2 did the FNAC bring out a suspicious picture of MTC. However, none of these tools specifically facilitated the identification of MTC in combination with PTC. The PTC/MTC association does not modify the epidemiological and pathological behavior and clinical characteristics of the cancer forms compared to their exclusive presentation; the prognosis depends mainly on the stage at the diagnosis of MTC [9]. However, the PTC/MTC form might display a more indolent behavior than the exclusive MTC because the MTC component tends to occur in a minor stage; in fact, according to a previous study, the percentage of deaths in the PTC/MTC form was 13.6% against 23.7% (*p* = 0.003) of exclusive MTC patients [15]. In our series, postoperative recurrence with MTC metastatic disease was detected in two patients (patient 4 and 5), with the highest stage at presentation (stage III and II, respectively). The clinical cases examined in the current paper agree with the majority of data reported to date in the literature (Table 2): (I) in our series, the mean age at diagnosis was of 55.4 years, with a range in the literature between 49.5 [9] and 64 [11], excluding the familial cases that occur at a younger age; (II) the prevalence of concurrent PTC in MTC patients was 20.8%, similar to the 19% detected by Kim et al. [13]; (III) mPTC represents the main method of presentation of DTC in PTC/MTC (80% in our patients, 81%, 75% and 77% in three previous Italian series [9,22], including the largest reported to date [3]), making MTC diagnosed generally before the other cancer subtype in this setting; interestingly, a large series of thyroid surgeries, performed because of benign disease (such as Graves’ disease of MNG), showed a much smaller rate of incidentally discovered mPTC (26–28%) [33]. On the contrary, the prevalence of the male gender (60%) diverges from all the other studies reviewed, which reported a female prevalence albeit with different values (from 60 to 100%), except for the study of Dionigi et al. [12], where, out of two cases, one patient was a woman and one a man. With this study, we aim to enhance the experiences about PTC/MTC, reporting detailed clinical and pathological features of the five identified cases. In fact, despite the long timeframe considered for our analysis, each single patient took a homogeneous diagnostic, therapeutic and follow-up path in line with available evidence, including the pre-operative measurement of calcitonin and CEA and research of any germline mutations for the RET gene, under the supervision of the same endocrinologist. Nevertheless, the paucity of available patients, even in a high-volume center for TC such as ours, restricts the chance of deeper research on this topic. Moreover, the retrospective nature of our investigation and, consequently, the lack of extensive molecular analysis of the cytological and pathological samples at the time of the diagnosis, represent further limits of this study in meeting the current need of a more personalized management, even if the analysis performed considered the exons that carry the most frequent mutations of both sporadic and germline mutations.

## 7. Conclusions

The simultaneous presence of papillary and medullary carcinoma in the same thyroid gland is a rare but noteworthy condition, whose incidence has been increasing over the past 30 years. The measurement of presurgical Ct is crucial for catching MTC, both when it is isolated and when concurrent to another TC such as PTC, and we strongly support this practice even if the cytology is already positive for PTC. The prognosis is mainly related to the stage of presentation and of MTC at diagnosis. Larger and prospective studies, with deeper molecular investigations, are needed to elucidate this still peculiar presentation of TC.

## Figures and Tables

**Table 1:** Baseline and clinical characteristics of our patients.

		Patient						PTC					MTC						
N°	Gender	Age (Years)	Family History of Thyroid Disease	1st Diagnosis	Other Thyroid Disease	Length of Follow-Up (Years)	Dimension (cm)	Lobe	Subtype	Stage	Molecular Mutation	Last Disease Status	Dimension (cm)	Lobe	Stage	RET Status	Pre-Operative Ct (pg/mL)	Last Ct (pg/mL)	Last Disease Status
1	M	59	Yes	MTC	CTL	15	0.2	Right	follicular	I	not investigated	NED	1.3	Left	III	negative	894	<2	biochemically cured
2	F	55	Yes	MTC	MNG	4	0.4	Left	classic	I	not investigated	NED	0.6	Left	I	negative	16.9	<2	biochemically cured
3	M	56	Yes	PTC	MNG	5	1.1	Left	classic	I	BRAF	NED	0.2	Right	I	negative	6.3	<2	biochemically cured
4	F	66	No	MTC	MNG	15	0.1	Left	not specified	I	not investigated	NED	2.8	Right	III	negative	478	1.8	biochemically cured
5	M	41	Yes	MTC	MNG	6	0.4	Right	not specified	I	not investigated	NED	4.7	Left	II	negative	2474	3295	loco-regional disease

Abbreviations: M, male; F, female; PTC, papillary thyroid carcinoma; MTC, medullary thyroid carcinoma; MNG, multinodular goiter; Ct, calcitonin (normal range: <11.5 pg/mL); NED, no evidence of disease.

**Table 2:** Studies published on PTC/MTC since 2000.

Study	Number of Cases	Average Age	Gender	RET Status	RET Mutated Codon	Other Molecular Mutations	Prevalence of mPTC
Behrend et al., 2002 [9]	1	61	M	N/A	N/A	N/A	100%
Biscolla et al., 2004 [10]	27	49.9	66% F	positive 5/27 (14%): all germinal	N/A	N/A	77%
Rossi et al., 2005 [5]	1 sporadic MTC	61	F	exon 11 (somatic)	C634R	BRAF V600E	N/A
	2 hereditary MTC	27 and 34	100% M	exon 15 (germinal)	both A891S	BRAF V600E	0%
Younes et al., 2005 [11]	1	55	F	N/A	N/A	N/A	N/A
Dionigi et al., 2007 [12]	2	49.5	50% F	1: negative 2: N/A	N/A	N/A	100%
Kim et al., 2010 [13]	10	53	60% F	N/A	N/A	N/A	90%
Costanzo et al., 2010 [14]	2	N/A	100% F	N/A	N/A	N/A	100%
Wong et al., 2012 [15]	162	53	64% F	N/A	N/A	N/A	N/A
Machens et al., 2012 [16]	26	50	54% F	positive in 6/26 (23%): all germinal	1 C634R 1 L709F 2 V804L 2 A891A	N/A	N/A (mean diameter 8 mm [3,4,5,6,7,8,9,10,11,12])
Verdi et al., 2012 [17]	1	64	M	negative	negative	N/A	100%
Adnan et al., 2013 [18]	4	52.25	100% F	negative	negative	N/A	100%
Erhamamci et al., 2014 [19]	4	53	75% F	N/A	N/A	N/A	50%
Fibbi et al., 2014 [20]	1	47	M	negative	negative	BRAF V600E	100%
Cheung et al., 2014 [21]	1	59	F	negative	negative	N/A	100%
Ciampi et al., 2016 [22]	24	64	75% F	positive 10/24 (42%) germinal 2/10 (20%)	1 germinal S918A, 1 germinal A804M; 8 somatic. M918T	1/24: BRAF V600E 1/24: H-RAS 1/24: K-RAS	78%
Roshini et al., 2017 [23]	11	27	F	negative	negative	N/A	N/A
Tang et al., 2017 [24]	1	50	F	N/A	N/A	N/A	100%
Gadong et al., 2019 [25]	1	38	F	exon 15 (germinal)	N/A	N/A	no
Appetecchia et al., 2019 [3]	183	56	58% F	evaluated 112/183: positive 24/112 (21%) negative 88/183 (48%) germinal 5/24 (21%) N/A 71/183 (39%)	see foot notes *	N/A	81%
Hellmann et al., 2020 [26]	1	34	M	negative	negative	negative	100%
Ziaolhagh et al., 2021 [27]	1	62	F	N/A	N/A	BRAF V600E	no
Abdullah et al., 2022 [28]	1	53	M	N/A	N/A	N/A	100%
Our study	5	55.4	60% M	negative	negative	1/5: BRAF V600E	80%

Abbreviations: M, male; F, female; N/A, not available; mPTC, papillary thyroid microcarcinoma * 8 (2 germinal) V804M; 2 (1 germinal) M918T; 2 (1 germinal) C602S; 1 germinal A883F; 1 C630T; 1 del631E; 1 E632_I638del; 1 D898_E901del; 1 S891A; 6 unkown.

## Data Availability

The authors confirm that the data supporting the findings of this study are available within the article.
